# History of Pregnancy Complications and the Risk of Ischemic Stroke in Young Women

**DOI:** 10.1212/WNL.0000000000214009

**Published:** 2025-08-06

**Authors:** Esmée Verburgt, Nina A. Hilkens, Jamie I. Verhoeven, Mijntje M.I. Schellekens, Merel S. Ekker, Esther M. Boot, Mayte E. van Alebeek, Paul J.A.M. Brouwers, Renate M. Arntz, Gert Van Dijk, Rob A.R. Gons, Ingeborg W.M. Van Uden, Tom Den Heijer, Julia van Tuijl, Karlijn F. de Laat, Anouk G. Van Norden, Sarah E. Vermeer, Marian S. Van Zagten, Robert J. Van Oostenbrugge, Marieke J.H. Wermer, Paul J. Nederkoorn, Henk Kerkhoff, Fergus A. Rooyer, Frank G. Van Rooij, Ido R. Van Den Wijngaard, Anil M. Tuladhar, Marleen M.H.J. van Gelder, Nel Roeleveld, Ralph R. Scholten, Frank-Erik De Leeuw

**Affiliations:** 1 Department of Neurology, Radboud Institute of Medical Innovation, Donders Institute for Brain, Cognition and Behavior, Nijmegen, the Netherlands;; 2 Department of Neurology, Gelre Hospital, Apeldoorn, the Netherlands;; 3 Medisch Spectrum Twente, Department of Neurology, Enschede, the Netherlands;; 4 Department of Neurology, Canisius-Wilhelmina Hospital, Nijmegen, the Netherlands;; 5 Department of Neurology, Catharina Hospital, Eindhoven, the Netherlands;; 6 Department of Neurology, Franciscus Gasthuis and Vlietland Hospital, Rotterdam, the Netherlands;; 7 Department of Neurology, Elisabeth-TweeSteden Hospital, Tilburg, the Netherlands;; 8 Department of Neurology, Haga Hospital, The Hague, the Netherlands;; 9 Department of Neurology, Amphia Hospital, Breda, the Netherlands;; 10 Department of Neurology, Rijnstate Hospital, Arnhem, the Netherlands;; 11 Department of Neurology, Jeroen Bosch Hospital, 's-Hertogenbosch, the Netherlands;; 12 Department of Neurology, Maastricht University Medical Centre, the Netherlands;; 13 Department of Neurology, Leiden University Medical Centre, the Netherlands;; 14 Department of Neurology, University Medical Centre Groningen, the Netherlands;; 15 Department of Neurology, Amsterdam University Medical Centre, the Netherlands;; 16 Department of Neurology, Albert Schweitzer Hospital, Dordrecht, the Netherlands;; 17 Department of Neurology, Zuyderland Hospital, Sittard-Geleen, the Netherlands;; 18 Medical Centre Leeuwarden, Department of Neurology, the Netherlands;; 19 Haaglanden Medical Centre, Department of Neurology, The Hague, the Netherlands;; 20 IQ Health Science Department, Radboud University Medical Center, Nijmegen, the Netherlands; and; 21 Department of Obstetrics and Gynecology and Physiology, Radboud University Medical Center, Nijmegen, the Netherlands.

## Abstract

**Background and Objectives:**

Pregnancy complications, such as hypertensive disorders of pregnancy (HDP), small for gestational age (SGA), preterm birth, gestational diabetes, stillbirth, and miscarriage, may increase the risk of ischemic stroke several years later. The aim of this study was to quantify the association between a spectrum of pregnancy complications and (the cause of) ischemic stroke in young women (<50 years).

**Methods:**

This study included 358 women (18–49 years) with first-ever imaging-proven ischemic stroke from the Observational Dutch Young Symptomatic StrokE study and 714 women from the Pregnancy and Infant Development Study, frequency-matched on age at most recent pregnancy and gravidity.

**Results:**

Women with an ischemic stroke at young age (median maternal age = 28 years [IQR = 24–31]) were more likely to have a history of HDP, SGA, preterm birth, gestational diabetes, stillbirth, and miscarriage, compared with women without ischemic stroke (median maternal age = 29 years [IQR = 26–31]). Specifically, the risk of having an ischemic stroke due to (likely) large artery disease, compared with women with a cryptogenic stroke, was increased for women with a history of HDP, SGA, and preterm birth.

**Discussion:**

A history of pregnancy complications may identify women at increased risk of ischemic stroke at young age, in particular atherosclerotic stroke.

## Introduction

The incidence of ischemic stroke at young age (<50 years) is higher among women than men.^1-3^ This may partly be explained by sex-specific risk factors, such as a history of pregnancy complications. These complications have found to be associated with an increased risk of ischemic stroke after the age of 50 years. One mechanism could be that pregnancy complications and ischemic stroke share similar endothelial and microvascular pathophysiology.^4^

Whether pregnancy complications are associated with specific etiologic subtypes of stroke at young age is of yet unknown. Investigating the relationship between pregnancy complications and etiologic subtypes might give insight into why these women have increased risk of stroke.

The aim of this study was to quantify the associations between pregnancy complications and (the cause of) ischemic stroke in young women.

## Methods

A case-cohort study was performed to quantify the associations between pregnancy complications (exposure) and ischemic stroke (outcome) at young age (18–49 years). Cases were included from the multicenter prospective cohort Observational Dutch Young Symptomatic StrokE studY (ODYSSEY).^5^ This study included consecutive patients from 17 different hospitals with a first-ever ischemic stroke between 18 and 49 years between May 2013 and February 2021, with follow-up until February 2024. Cause of stroke was based on the modified Trial of Org 10172 in Acute Stroke Treatment (TOAST) classification.^6^ For every case with an ongoing pregnancy, 2 controls were frequency matched from the nationwide Pregnancy and Infant Development (PRIDE) Study. The PRIDE Study is an ongoing prospective cohort study investigating factors affecting the health of the mother and child during and after pregnancy. Since 2011, women aged 18 years or older in early pregnancy are included, with follow-up for each mother until their child reaches the age of 21 years.^7^

Pregnancy complications included a miscarriage at any time <20 weeks of gestation, gestational hypertension, preeclampsia, small for gestational age (SGA) (birth weight <10th percentile, according to reference curves based on Perined [Dutch Perinatal Registry]), preterm birth (<37 weeks), gestational diabetes, and stillbirth (≥20 weeks).^8^ hypertensive disorders of pregnancy (HDP) was defined as gestational hypertension and/or preeclampsia in line with criteria set by the International Society for the Study of Hypertensive in Pregnancy.^9^ We also reported the occurrence of HDP in multiple pregnancies. The eMethods provides details on exclusion criteria of cohorts and ascertainment of pregnancy complications.

Both studies were approved by the local ethics committee “East-Netherlands,” and all participants provided written (ODYSSEY) or digital (PRIDE Study) informed consent.

To assess the associations between a history of pregnancy complications in any pregnancy and ischemic stroke, odds ratios (ORs) with 95% CIs were estimated using logistic regression while adjusting for maternal age at first pregnancy. A sensitivity analysis was performed for pregnancy complications in only the first pregnancy. Multinomial logistic regression analyses were used to estimate ORs for the associations between pregnancy complications and cause of ischemic stroke, also adjusted for maternal age at first pregnancy.

## Results

A total of 358 women with ischemic stroke with 736 previous pregnancies and 714 women without ischemic stroke with 1,431 pregnancies were included in this study (eFigure 1). Median gestational age at delivery of the first child was comparable between women with and without ischemic stroke, as given in Table 1. The birth weight of the first child was lower in women with ischemic stroke. eTable 1 summarizes the prevalence of cardiovascular risk factors per cohort. The median interval between first pregnancy and stroke was 16 years.

**Table 1:** Characteristics of Women With and Without an Ischemic Stroke

	Women with ischemic stroke, n = 358	Women without ischemic stroke, n = 714
N of pregnancies, median (IQR)	2 (2–2)	2 (1–2)
Maternal age first pregnancy in y, median (IQR)	28 (24–31)	29 (26–31)
Gestational age at delivery first child in wk, median (IQR)	39 (37–40)	39 (38–40)
Birth weight first child in grams, median (IQR)	3,190 (2,695–3,665)	3,400 (3,050–3,740)

Abbreviation: IQR = interquartile range.

Missing for women with ischemic stroke: maternal age at first pregnancy 4 (1.1%), gestational age 73 (20.4%), birth weight 91 (25.4%). Missing for women without ischemic stroke; maternal age at first pregnancy 1 (0.1%), gestational age 22 (3.1%), birth weight 67 (9.4%).

### Pregnancy Complications in Women With or Without Ischemic Stroke

One hundred eighty-two of 352 (50.8%) women with ischemic stroke had experienced 1 or more pregnancy complications, compared with 219 of 714 (30.7%) women without ischemic stroke (Table 2). Pregnancy complications that were associated with ischemic stroke at young age were HDP, HDP in multiple pregnancies, preeclampsia, SGA, preterm birth, gestational diabetes, stillbirth, and miscarriage. This was similar for pregnancy complications that occurred during the first pregnancy only (eTable 2).

**Table 2:** Pregnancy Outcomes in Any Pregnancy in Women With and Without Ischemic Stroke

	Women with ischemic stroke, n = 358	Women without ischemic stroke, n = 714	OR (95% CI)^a^
Complication in any ongoing pregnancy, n (%)^b^	182 (50.8)	219 (30.7)	2.3 (1.8–3.0)
HDP, n (%)	89 (24.9)	103 (14.4)	2.0 (1.4–2.7)
Pregnancy-induced hypertension, n (%)^c^	46 (12.8)	85 (11.9)	0.9 (0.6–1.4)
Preeclampsia, n (%)^c^	43 (12.0)	24 (3.4)	4.0 (2.4–6.8)
HDP in multiple pregnancies, n (%)	37 (10.3)	20 (2.8)	3.9 (2.2–6.9)
SGA, n (%)	88 (24.4)	77 (10.8)	2.8 (2.0–3.9)
Preterm birth, n (%)	70 (19.4)	57 (8.0)	2.7 (1.9–4.0)
Gestational diabetes, n (%)	19 (5.3)	16 (2.2)	2.6 (1.3–5.3)
Stillbirth, n (%)	12 (3.4)	4 (0.6)	4.8 (1.6–17.8)
Miscarriage, n (%)	120 (33.5)	203 (28.4)	1.3 (1.0–1.8)

Abbreviations: HDP = hypertensive disorders of pregnancy; OR = odds ratio; SGA = small for gestational age (<10th birth centile).

a Adjusted for maternal age at first pregnancy.

b Any (ongoing) pregnancy complication is defined as HDP, SGA, preterm birth, gestational diabetes, and/or stillbirth.

c If a woman had gestational hypertension in 1 pregnancy and preeclampsia in another, she is counted in both groups.

### Pregnancy Complications and Etiologic Subtypes of Ischemic Stroke

Women with an ischemic stroke at young age due to (likely) large artery disease were more likely to have a history of HDP, HDP in multiple pregnancies, SGA, or preterm birth, compared with women with a cryptogenic stroke (Table 3).

**Table 3:** Pregnancy Outcomes in Any Pregnancy Per Cause of Stroke

	(Likely) large artery disease^a^, n = 71	Small vessel disease, n = 43	Other causes, n = 141	Cryptogenic, n = 103
Complication in any ongoing pregnancy, n (%)^b^	50 (70.4)	24 (55.8)	62 (44.0)	45 (43.7)
OR (95% CI)^c^	3.1 (1.6–5.9)	1.6 (0.8–3.4)	1.0 (0.6–1.7)	1 (reference)
HDP, n (%)	29 (40.8%)	10 (23.3%)	29 (20.6%)	21 (20.4%)
OR (95% CI)^c^	3.0 (1.5–6.1)	1.3 (0.5–3.1)	1.1 (0.6–2.1)	1 (reference)
HDP in multiple pregnancies, n (%)	14 (19.7)	5 (11.6)	10 (7.1)	8 (7.7)
OR (95% CI)^c^	3.4 (1.3–9.1)	1.8 (0.5–6.1)	1.1 (0.4–2.9)	1 (reference)
SGA, n (%)	25 (35.1)	9 (12.5)	32 (22.7)	22 (21.4)
OR (95% CI)^c^	1.9 (1.0–3.8)	0.9 (0.4–2.2)	1.1 (0.6–2.0)	1 (reference)
Preterm birth, n (%)	21 (29.6%)	11 (26.2%)	20 (14.2%)	18 (17.5%)
OR (95% CI)^c^	2.1 (1.0–4.4)	1.7 (0.7–4.1)	0.8 (0.4–1.6)	1 (reference)
Gestational diabetes, n (%)	5 (7.0)	6 (14.0)	3 (2.2)	5 (4.9)
OR (95% CI)^c^	1.5 (0.4–5.4)	3.2 (0.9–11.2)	0.4 (0.1–1.9)	1 (reference)
Stillbirth, n (%)	3 (4.2)	2 (4.7)	4 (2.8)	3 (2.9)
OR (95% CI)^c^	2.0 (0.3–12.6)	2.2 (0.3–16.4)	1.0 (0.2–6.4)	1 (reference)
Miscarriage, n (%)	20 (28.2)	12 (27.9)	52 (36.9)	36 (35.0)
OR (95% CI)^c^	0.7 (0.4–1.3)	0.6 (0.3–1.4)	1.1 (0.6–1.8)	1 (reference)

Abbreviations: HDP = hypertensive disorders of pregnancy; OR = odds ratio; SGA = small for gestational age.

a (Likely) Large artery disease is composed of the TOAST classification “large artery disease” (n = 9) and “likely large artery disease” (n = 62).

b Any (ongoing) pregnancy complication is defined as HDP, SGA, preterm birth, gestational diabetes, and/or stillbirth.

c Adjusted for maternal age at first pregnancy.

## Discussion

We found that women with ischemic stroke at young age were more likely to have a history of HDP, HDP in multiple pregnancies, SGA, preterm birth, gestational diabetes, stillbirth, and miscarriage compared with women without ischemic stroke. In addition, young women with ischemic stroke due to (likely) large artery disease more often had a history of HDP, HDP in multiple pregnancies, SGA, and preterm birth, compared with a cryptogenic stroke. Almost half of the women with an otherwise cryptogenic stroke had a history of 1 or more pregnancy complications in any ongoing pregnancy.

A unique contribution of our study is phenotypical details of cause of stroke, which may reveal some information about possible shared mechanisms. Ischemic stroke and pregnancy complications are believed to share atherosclerotic pathophysiology, such as endothelial dysfunction, and (micro)vascular dysfunction.^4^ Women with atherosclerotic disease are more likely to have abnormal placental vascularization, resulting in ischemic placental disease with subsequent pregnancy complications.^4^ One hypothesis is that pregnancy complications could be a first manifestation of cardiovascular disease in women, such as ischemic stroke. The other hypothesis is that pregnancy complications promote atherosclerosis, contributing to the excess ischemic stroke risk.^4^ Irrespective of the causal pathway, we found that HDP, HDP in multiple pregnancies, SGA, and preterm birth were associated with (likely) large artery disease as the cause of stroke, indicating later-in-life atherosclerotic disease in these patients.

Despite pregnancy complications being associated with ischemic stroke due to (likely) large artery disease in young women with ischemic stroke, more than 40% of women with cryptogenic stroke also had 1 or more pregnancy complications in their medical history, possibly hinting toward an atherosclerotic mechanism behind some of these cryptogenic strokes.

Our findings have important clinical implications. First, we emphasize that pregnancy complications need to be recognized as a potential indicator of increased cardiovascular risk, including stroke at young age, and should be evaluated in clinical care. Second, these findings should prompt clinicians to optimize clinical management in these women. Thus far, the international guidelines on secondary cardiovascular risk management after pregnancy complications, such as the Dutch Society of Obstetrics and Gynecology, suggest cardiovascular risk management in women with preeclampsia after the age of 50.^10^ Our study supports cardiovascular risk management in these women even at a younger age; however, future studies should investigate the effects of lifestyle modification aimed at reducing cardiovascular risk in women with adverse pregnancy outcomes.

Some limitations need to be addressed. First, the histories of pregnancy complications were entirely self-reported in the ODYSSEY and partly self-reported in the PRIDE Study, potentially resulting to overestimation or underestimation of the occurrence of pregnancy complications. However, previous studies reported that the self-reported occurrence of pregnancy complications is generally good, with the exception of overestimation of pregnancy-induced hypertension.^11,12^ Second, participants in the PRIDE study are recruited prospectively, meaning we cannot rule out that some of these women will have developed ischemic stroke at young age. We expect this risk to be low because ischemic stroke at young age is rare. Finally, we could not adjust for the confounding cardiovascular risk factors in statistical analyses because they were assessed at time of ischemic stroke in the ODYSSEY study and at time of last pregnancy in the PRIDE Study.

In conclusion, we found that a history of pregnancy complications is associated with an increased risk of ischemic stroke and more specifically atherothrombotic stroke.
